# The Association between Attention-Deficit–Hyperactivity Disorder and Autistic Traits with Psychotic-like Experiences in Sample of Youths Who Were Referred to a Psychiatric Outpatient Service

**DOI:** 10.3390/brainsci14080844

**Published:** 2024-08-22

**Authors:** Laura Fusar-Poli, Chiara Avanzato, Giuliana Maccarone, Elide Di Martino, Gabriele Avincola, Stefania Grasso, Giovanni Rapisarda, Francesco Guarnieri, Maria Salvina Signorelli

**Affiliations:** 1 Department of Brain and Behavioral Sciences, University of Pavia, 27100 Pavia, Italy; laura.fusarpoli@unipv.it; 2 Psychiatry Unit, Department of Clinical and Experimental Medicine, University of Catania, 95123 Catania, Italy; chi4493b@gmail.com (C.A.); giuliana.mcc@gmail.com (G.M.); elidedimartino@gmail.com (E.D.M.); avincolag@gmail.com (G.A.); stefaniagrasso96@gmail.com (S.G.); 3 Territorial Unit of Child and Adolescent Neuropsychiatry, 95125 Catania, Italy; grapisarda@aspct.it (G.R.); francesco.guarnieri64@gmail.com (F.G.)

**Keywords:** adolescence, autism spectrum disorder (ASD), attention-deficit–hyperactivity disorder (ADHD), traits of a disorder, subthreshold symptoms, psychotic-like experiences (PLEs)

## Abstract

The aim of this study is to identify autism spectrum disorder (ASD) and attention-deficit–hyperactivity disorder (ADHD) traits in adolescents who experience psychotic-like experiences (PLEs), often ignored in clinical practice but widely prevalent in the general population. A total of 57 adolescents and young adults (aged between 16 and 24 years old) were recruited consecutively in the outpatient services. A total of 37 were females (64.9%), 18 were males (31.6%), and two participants (3.5%) were non-binary or gender non-conforming, with a mean age at referral of 18.26 ± 2.06. To investigate these symptoms, three sets of standardized questionnaires were used, as follows: the Autism Spectrum Quotient–short form (AQ-10), the Community Assessment of Psychic Experiences (CAPE-42), and the Adult ADHD Self-Report Scale (ASRS). We found significant associations between the ASRS and AQ-10 total scores and all CAPE scales. The model which explained the highest variance was CAPE Score’s Total Frequency score (*p* < 0.001). Our findings underline the importance of investigating the presence of subthreshold ASD and ADHD symptoms in clinical populations, particularly in the period of adolescence and young adulthood, to promptly identify the presence of PLEs and, thus, prevent the onset of a frank psychotic disorder, particularly in the presence of a comorbid psychopathological condition, leading to better diagnosis and treatment for individuals with two or more of these conditions.

## 1. Introduction

Adolescence, spanning ages 10 to 24, is a crucial developmental phase marked by significant biological, psychological, and social changes that bridge childhood and adulthood [1]. According to the United Nations Population Fund (UNFPA) [2], there are approximately 1.2 billion individuals aged 10 to 19 worldwide, accounting for about 16% of the global population, while people aged 20 to 24 make up around 10% of the global population. This period is characterized by rapid physical growth, cognitive development, and the onset of puberty, all of which contribute to the formation of a more complex identity and a deeper sense of self. As such, adolescence is considered a vulnerable phase that is essential for laying the groundwork for long-term health.

Recent meta-analytic research [3] underscores the impact of significant biological changes in the brain during neurodevelopmental phases. Throughout the transition from childhood to adulthood, the brain undergoes notable transformations. Grey matter volume increases during childhood, peaks around puberty, and then declines, particularly in the frontal and parietal lobes, due to synaptic pruning. In contrast, white matter volume continues to expand throughout childhood and adolescence, potentially reaching its peak in the third decade of life, driven by myelination and possible changes in axon thickness. Functional connectivity between brain regions is enhanced with age, supporting cognitive functions such as memory. Additionally, adolescents exhibit altered brain activity in regions associated with reward processing, which may account for increased risk-taking behaviours [4]. These developmental changes are crucial for understanding both typical brain maturation and the emergence of psychiatric disorders. The meta-analysis reveals that the proportion of individuals who develop psychiatric disorders before age 25 is notably high: over 90% for autism spectrum disorder (ASD) and attention-deficit/hyperactivity disorder (ADHD), and 40–50% for schizophrenia, with minimal differences between males and females.

Recognizing the traits of such disorders is important for prevention or early diagnosis. According to the dimensional approach, a trait is not simply present or absent but is instead studied along a continuum. In this context, the modern literature has introduced the concept of the spectrum for certain disorders, such as autism spectrum disorder (ASD), attention-deficit–hyperactivity disorder (ADHD), and the schizophrenia spectrum [5]. Based on this concept, traits can be present in the general population on a continuum of severity and intensity. Specifically, atypical social communication and behavioural inflexibility are some of the principal traits of ASD; features of ADHD include impaired levels of attention, disorganization, and/or hyperactivity [6], while the psychotic-like experiences (PLEs) are milder manifestations of psychotic symptoms characterized by transient and attenuated perceptual and thought disturbances compared to overt symptoms such as hallucinations and delusions [7]. Moreover, considering the continuum model of disturbances, it has been seen that ASD and ADHD traits have been associated with PLEs [8]. The identification of these characteristics during adolescence may enable better diagnosis and treatment for individuals with two or more of these conditions.

The diagnosis of ASD is complex and typically involves multiple steps, as it encompasses a wide range of symptoms. According to the last version of the *Diagnostic and Statistical Manual of Mental Disorders, 5th edition, Text Revision* (DSM 5-TR) [5], the criteria for ASD include specific patterns of behaviour and difficulties in social communication. To be diagnosed with ASD, persistent deficits in social communication and social interaction and restricted, repetitive patterns of behaviour, interests, or activities must be present. Symptoms usually emerge in early childhood but might not become fully noticeable until social demands surpass the individual’s abilities or are concealed by coping strategies developed later in life. Additionally, some specifiers can help clarify the clinical picture of the patient by outlining the severity level of the disorder or the presence of language or intellectual impairments. As mentioned, the complexity of the diagnosis necessitates several steps for a correct diagnostic assessment. Detailed interviews with parents or caregivers about the child’s behaviour, development, and family history, along with direct observation of the child’s behaviour across different settings, can already guide toward a clinical diagnosis. To refine the clinical diagnosis, some autism-specific diagnostic tools have been developed: Autism Diagnostic Observation Schedule (ADOS) [9,10], a structured play-based assessment that evaluates social interaction, communication, and play, and the Autism Diagnostic Interview—Revised (ADI-R) [11,12], a structured interview with parents that focuses on the child’s developmental history and behaviour. The DSM-5 consolidated previous autism subcategories (such as Asperger’s Syndrome) into a single spectrum, broadening the criteria and potentially increasing the number of individuals diagnosed, which may contribute to overdiagnosis. Tools like the ADI-R and ADOS are commonly used, but their effectiveness hinges on the clinician’s skill and experience. Accurate scoring and interpretation of these tools require substantial clinical judgement, and without it, overdiagnosis can occur. While evidence of overdiagnosis is mostly indirect and anecdotal, it is suggested by high ASD prevalence rates and a significant number of community-diagnosed cases failing to meet strict research criteria upon reassessment. Overdiagnosis can have serious consequences, such as restricting individuals’ social and educational opportunities and impacting their identity development. It can also create inequities in access to services, diverting resources from children with other neurodevelopmental disorders. To prevent overdiagnosis, it is crucial to use multiple data sources, track symptom progression, and ensure that any functional impairments are specifically linked to autism rather than other conditions. Last but not least, clinicians should adhere to validated procedures when integrating diagnostic information, with a strong emphasis on clinical judgement and expertise.

The same diagnostic complexity is found in ADHD. It requires a comprehensive approach that includes detailed behavioural evaluations, input from multiple sources, and the application of DSM-5 TR criteria. To be diagnosed, a persistent pattern of inattention and/or hyperactivity–impulsivity must be present and must have arisen before the age of 12 [5]. An individual’s medical history, including any relevant family history, prenatal and birth history, and developmental milestones, as well as observations of the individual’s behaviour in various settings, such as at home, school, or work, are crucial for the clinical diagnosis. Teachers, parents, or caregivers are often asked to provide detailed accounts of the individual’s behaviour. Similarly, some specifiers can help provide more details about the clinical picture by indicating its severity, the predominance of inattention or hyperactivity/impulsivity, as well as the combined presentation. To refine the diagnosis, structured interviews with the patient and others who are familiar with their behaviour, such as parents, teachers, or employers, can be used, such as the following: Conners’ Rating Scales [13], the Adult ADHD Self-Report Scale (ASRS-V1.1) [14,15,16], and others.

As mentioned, PLEs are characterised by symptoms that are analogous to those observed in psychotic disorders, such as schizophrenia, but do not satisfy the full diagnostic criteria for such conditions. These experiences may involve hallucinations, delusional ideation, and disorganised thought processes; however, they are generally of lower severity, occur less frequently, and do not result in significant functional impairment. PLEs are often transient in nature and may be precipitated by external factors, including stress, sleep deprivation, or substance use [7].

A variety of pharmacological and non-pharmacological therapies have been proposed for these disorders, including Cognitive Behavioral Therapy (CBT), which helps individuals to identify and change negative thought patterns and behaviours, thus developing coping strategies and improving social skills [17]. CBT has also demonstrated moderate efficacy for anxiety disorders in youth with high-functioning ASD and for addressing persecutory ideation symptoms associated with PLEs [18,19]. Mindfulness practices, such as meditation and deep breathing exercises, can help reduce anxiety in patients with PLEs and improve attention and self-regulation in patients with ADHD or ASD traits [20,21]; regular exercise and physical activity have been shown to improve executive function, attention, and behaviour in individuals with ADHD [22]. Pharmacological interventions such as antipsychotic medications are often used to manage PLEs, especially when they progress to full psychotic symptoms; risperidone and aripiprazole are FDA-approved medications for treating irritability associated with ASD, alpha-2 adrenergic agonists (like clonidine and guanfacine) have shown some efficacy in reducing hyperactivity and stereotypic behaviours in children with ASD and ADHD [23,24].

It is important to note that medication should be used as part of a comprehensive treatment plan that includes behavioural interventions and therapy. Educators and families also play crucial roles in supporting children with autism. Teachers and school staff are responsible for creating inclusive and supportive learning environments. They can detect early traits of autism, provide appropriate interventions, and implement individualised education plans. Families, on the other hand, are advocates for their children, providing emotional support and participating in their education. They work closely with educators to share information, set goals, and ensure consistency between home and school environments [25,26].

Based on this evidence, the research questions were as follows:

Q1: Is there a correlation between PLEs and traits of ADHD? What is the nature of their relationship?

Q2: Is there a correlation between PLEs and traits of ASD? What is the nature of their relationship?

We hypothesize that, consistent with existing studies in the literature, there is a significant relationship between PLEs and traits of ASD and ADHD.

## 2. Materials and Methods

### 2.1. Study Design and Participants

We conducted a cross-sectional observational study between December 2019 and November 2022. A total of 57 adolescents and young adults (aged between 16 and 24 years old) were recruited consecutively in the outpatient services of the psychiatric unit of Policlinico “G. Rodolico” and from the Service of Child and Adolescent Psychiatry of the “Cittadella dell’Adolescenza” in Catania (Italy).

Participants were included in the present study if they fulfilled the following inclusion criteria: (1) aged between 16 and 24 years old; (2) first-time help-seeker without psychiatric diagnosis and psychopharmacological therapy. Exclusion criteria were the following: (1) neurological disorders, including a history of brain injury; (2) presence of intellectual disability; (3) past or present history of drug abuse, including alcohol and cannabis.

Before data collection, a preliminary interview was conducted with all patients who met the inclusion criteria. Diagnoses were made according to the criteria of the DSM-5-TR. Following this, the survey was administered using a Google Form on our computer tablets. To avoid missing data, the platform required users to complete all questions in a section before proceeding to the next. The survey was designed to be completed in less than 20 min. Participants did not receive any compensation for their participation.

The survey consisted of three sections. The first section included information about the project, the aim of the study, informed consent, and the researchers’ contacts. The second section of the survey included socio-demographic questions (i.e., age, sex, educational level, marital status, employment, and living status). The last section of the survey included three sets of standardized questionnaires: the Autism Spectrum Quotient–short form (AQ-10), the Community Assessment of Psychic Experiences (CAPE-42), and the Adult ADHD Self-Report Scale (ASRS). All of the data were collected anonymously and voluntarily. All participants provided written informed consent to participate in the studies and to have their survey results published. The study was conducted in accordance with the Declaration of Helsinki and approved by the Ethics Committee of University of Catania (2019/No. 1). It was approved on 14 January 2019.

### 2.2. Instruments

#### 2.2.1. The Autism Spectrum Quotient—10 Items (AQ-10)

The AQ scale was developed to test the hypothesis of autism as a trait with a continuous distribution in the general population [27] but is currently suggested as a screening tool for ASD by international guidelines [28]. Originally, the test included a total of 50 items in 5 subscales (AQ-50). Shortened versions with 28 items (AQ-Short) and 10 items (AQ-10) have been developed later. Test–retest and interrater reliability of the AQ was good. Cronbach’s alpha coefficients were all moderate to high (Communication = 0.65; Social = 0.77; Imagination = 0.65; Local Details = 0.63; Attention Switching = 0.67).

Each item is phrased as a statement for which the respondent rates the degree of agreement or disagreement on a four-point Likert response alternative. It consists of 10 statements with potential answers for each statement: definitely agree, slightly agree, slightly disagree, definitely disagree. One point is given directly if the participant’s answer agrees or slightly agrees or in reverse scoring. The total score can range between 0 and 10. A cut-off of ≥6 is suggestive for ASD.

#### 2.2.2. The Community Assessment of Psychic Experiences (CAPE-42)

The CAPE-42 is a self-administered 42-item questionnaire which aims to better determine the frequency of specific ideas, experiences, and psychic phenomena. It is based on the assumption that these ideas, experiences, and phenomena are also common in non-psychiatric samples.

The CAPE, derived from a combination of the Peters Delusions Inventory (PDI) [29], modified in the formulation of some items with the addition of items from the Scale for the Assessment of Negative Symptoms (SANS) [30] and the Subjective Experience of Negative Symptoms Scale (SENS) [31].

The items cover three dimensions: positive symptoms, depressive symptoms, and negative symptoms. Specifically, the CAPE includes 20 questions on positive symptoms, 14 on negative symptoms, and 8 on depressive symptoms. For each item, the frequency of individual experiences is indicated (possible four levels: never = 1, sometimes = 2, often = 3, always = 4) and, in the case of a positive response (score > 1), also the extent of the distress experienced, i.e., as formulated in the questionnaire, how much that particular experience ‘makes you feel sick’ (possible four levels: not at all = 1, a little = 2, enough = 3, a lot = 4). For each dimension, it is, therefore, possible to calculate two different total scores: frequency and distress. To take the possible non-response into account, the scores are weighted by the number of valid responses given for each dimension (i.e., the sum of the scores divided by the number of items to which a response was given).

The CAPE is an instrument with high sensitivity and, therefore, a low false-negative rate or high specificity and positive predictive power with a low number of false positives. Internal consistency, as assessed through Cronbach’s alpha coefficients, is found to be good, with coefficients ranging from 0.78 to 0.86 in the total sample in its Italian version validation [32,33].

A cut-off value of 2.80 for the positive dimension identifies individuals at very high risk (UHR—high-risk mental status) in a clinical population with high sensitivity (83%), although this is not sufficient for a diagnosis of UHR, expressing a potential risk of psychosis [34].

#### 2.2.3. The Adult ADHD Self-Report Scale (ASRS)

The ASRS is used as a screening tool for ADHD in the general population [14]. In the present study, we used the ASRS v.1.1 based on the DSM-5 criteria [15,16]. The subject is asked to provide responses regarding the frequency of 18 clinical manifestations in the past 6 months, ranging from “Never” to “Very often” (5 possibilities in total). For each question, although there are 5 choices, the scoring is binary (0 or a 1) depending on the presence of the symptom examined and its frequency. The scale covers two sets of questions, divided into two parts. “Part A” includes six questions which are considered the most reliable in predicting adult ADHD. If four or more positive answers for this part are given, the subject is considered at high risk of adult ADHD. “Part B” includes 12 further items which are considered to provide additional evidence that can be useful in a clinical context. In the present study, we used the total number of symptoms scaled, namely the sum of “Part A” and “Part B”, which can vary from 0 to 18, as it has been validated. It showed good reliability with Cronbach’s alpha coefficient of 0.71 (inattention), 0.62 (hyperactivity/impulsivity), and 0.76 (total score).

### 2.3. Statistical Analysis

All variables were tested for normal distribution using the Shapiro–Wilk test before statistical procedures were applied. Socio-demographic and clinical variables were reported as means and standard deviations (SD) if continuous and as counts and percentages if categorial. To evaluate the association between ADHD, autistic traits, and PLEs in our sample, we performed eight linear regression analyses, corrected by age and gender, using the ASRS and AQ-10 total score as the dependent variables and the CAPE-42 total, positive, depressive, and negative scores (both frequency and distress) as independent variables. Results were regarded as statistically significant for *p* < 0.05. Statistical analyses were performed using SPSS 24.0.

## 3. Results

### 3.1. Characteristics of Participants

We recruited a total of 57 patients, of which 37 were females (64.9%) and 18 were males (31.6%). Two participants (3.5%) were non-binary or gender non-conforming. The mean age at referral was 18.26 ± 2.06, with ages ranging from 16 to 23 years. Regarding the highest educational level achieved, 32 (56.1%) had completed middle school, while 25 (43.9%) had obtained a high school diploma. Moreover, 77.2% of the sample was composed of students, 7% of workers, 8.8% of students/workers, and another 7% were unemployed. Only 6 participants (10.5%) were living alone, while the majority were living with their families (89.5%). None of the participants were married, nor did they have offspring. All socio-demographic characteristics of the sample are reported in Table 1.

### 3.2. Clinical Characteristics

After the clinical evaluation, 37 participants (64%) received a diagnosis of anxiety disorder. Mood disorders were diagnosed in nine participants (15.8%). Eleven participants (19.3%) were diagnosed with a personality disorder. Table 2 presents the scores reported by participants on the ASRS and AQ-10 total scores, as well as the CAPE total frequency and distress, CAPE positive frequency and distress, CAPE negative frequency and distress, and CAPE depressive frequency and distress.

### 3.3. Association of Autistic and ADHD Traits with Psychotic-like Symptoms

Linear regressions were used to assess how the Adult ADHD Self-Report Scale (ASRS) and Autism Spectrum Quotient–short form (AQ-10) total scores correlate with all subscales of the Community Assessment of Psychic Experiences (CAPE). To ensure that the identified associations were independent of other confounding variables, age and gender were added as covariates to the models, showing no impact on the analysis.

Our analysis revealed significant associations between both ASRS and AQ-10 total scores and all subscales of the CAPE. These associations were observed across both the frequency and distress dimensions of the CAPE subscales.

The strongest association was found between ASRS scores and the CAPE Total Frequency score (β = 0.47, 95% CI: 0.06–0.18, *p* < 0.001), with this model explaining the highest variance (R^2^ = 0.49) among all tested relationships. This suggests that ADHD symptoms are most strongly related to the overall frequency of psychotic-like experiences.

Notably, both ASRS and AQ-10 showed consistent positive associations with all CAPE subscales, indicating that higher levels of ADHD and autistic traits are associated with increased frequency and distress of psychotic-like experiences. The strengths of these associations varied across different CAPE dimensions, with beta coefficients ranging from 0.09 to 0.55 for ASRS and from 0.26 to 0.46 for AQ-10.

Interestingly, the associations were generally stronger for the frequency of psychotic-like experiences compared to the distress they caused, particularly for the AQ-10 scores. This pattern was consistent across total, positive, depressive, and negative symptom dimensions of the CAPE.

These findings underscore the complex interplay between ADHD symptoms, autistic traits, and psychotic-like experiences, suggesting potential shared underlying mechanisms or risk factors across these neurodevelopmental and psychiatric domains.

Table 3 shows the results of the linear regressions between ASRS, AQ-10, and CAPE.

## 4. Discussion

The aim of this study was to evaluate the association between autistic and ADHD traits and PLEs in a group of adolescents and young adults who were referred—for the first time—to a specialized service in Italy. Our analysis revealed significant associations between both the Adult ADHD Self-Report Scale (ASRS) and Autism Spectrum Quotient–short form (AQ-10) total scores and all subscales of the Community Assessment of Psychic Experiences (CAPE). These associations were observed across both the Frequency and Distress dimensions of the CAPE subscales.

The significant associations between the ASRS and AQ-10 total scores and all CAPE scales we found are in line with previous studies, confirming that both ADHD and ASD traits are independently associated with PLEs. As for the association between PLEs and ASD, a meta-analysis conducted by Kiyono et al. [35] suggests that the prevalence of PLEs among individuals with ASD is higher (24%) than in the general population (5–8%), even if PLEs subtypes have different prevalence (e.g., hallucinations, 6%; delusions, 45%). Nevertheless, to date, the reason for this association has yet to be clarified.

Analogously, the association between PLEs and ADHD has been underlined by several studies [7,36]. One potential reason is that ADHD may increase the risk of traumatic experiences in childhood, making PLEs more likely [37]. At the same time, subjects who experience PLEs, who are consequently at high risk of developing full-blown symptoms, show attention deficits in adolescence. These deficits are more emphasized in divided-attention tasks (capacity to reply concurrently to quite a few tasks) that need more attention than in sustained-attention tasks (capacity to maintain a steady behavioural reaction during a repeating task) [7].

Additionally, adverse childhood experiences, such as peer victimization, social rejection or isolation, and stress from academic failure, are common in individuals with ASD [38] and ADHD [39]. Such stressors might increase the risk of developing PLEs [40,41].

Although the literature has consistently found associations between PLEs and both clinical ASD [8] and ADHD [7,42], research on subthreshold symptoms, which are the focus of our study, is still underdeveloped. It has been hypothesized that individuals with higher ASD or ADHD traits may be at a greater risk of developing mental disorders than people with lower ASD and ADHD traits [43,44]. For instance, we have recently shown that these traits predict Internet gaming disorder in adults [45]. It is worth considering that PLEs are familial and heritable, have an increased risk of schizophrenia spectrum disorders, covary with maternal schizophrenia-spectrum disorder, and share a wide range of risk factors with schizophrenia [46]. People with PLEs are more likely to develop psychotic, affective, anxiety, behavioural, and substance use disorders [47]. Additionally, it has been shown that the co-presence of PLEs and non-psychotic disorders increases the risk of transition to psychosis more than PLEs alone [48].

Our data underscore the importance of investigating subthreshold ASD and ADHD traits in clinical populations, especially during adolescence and young adulthood, to promptly identify the presence of PLEs and prevent the onset of overt psychotic disorders, particularly in individuals with comorbid psychopathological conditions. Clinicians working with individuals who exhibit subthreshold ASD or ADHD symptoms should be alert to the potential presence of PLEs, as these may indicate an increased risk for developing more severe mental health conditions, including psychotic disorders. The findings suggest that integrating assessments for PLEs into routine evaluations of young people with ASD or ADHD traits could lead to earlier identification of those at higher risk, potentially allowing for more timely and targeted interventions. Furthermore, this research opens up new avenues for investigating the complex interplay between neurodevelopmental traits and psychotic experiences, which could ultimately lead to improved prevention strategies and treatment approaches for a range of mental health conditions.

## 5. Limitations

The study has several limitations. Firstly, the sample size is limited; however, as this is a pilot study, we plan to recruit a larger number of participants in the future. Secondly, the AQ-10 and CAPE-42 are self-reported questionnaires and may be less accurate than clinician-rated interviews due to recall bias or limited patient awareness. Thirdly, our analysis was conducted with only an outpatient sample of autistic adolescents and adults, so the findings may not be generalizable to other populations. Fourthly, we have not investigated potential associations between PLEs and the medications taken by patients for their comorbid conditions, nor have we examined the use of birth control medications. Moreover, although we identified a history of drug abuse, including alcohol and cannabis, as an exclusion criterion, we have not examined the use of tobacco, whether smoked or vaped. Finally, as this is a cross-sectional study, we cannot establish cause-and-effect relationships.

## 6. Conclusions

This study provides valuable insights into the relationship between autistic traits, ADHD traits, and psychotic-like experiences (PLEs) in adolescents and young adults. The significant associations found between these traits and PLEs underscore the complexity of neurodevelopmental and psychiatric symptoms in this population. Our findings suggest that individuals with higher levels of autistic and ADHD traits may be at increased risk for experiencing PLEs, which in turn could potentially lead to more severe mental health outcomes.

The results highlight the importance of comprehensive assessment and early intervention strategies in clinical settings. Clinicians working with young people should be aware of the potential co-occurrence of autistic traits, ADHD traits, and PLEs and consider screening for these experiences, even in individuals who do not meet full diagnostic criteria for ASD or ADHD.

While this pilot study has limitations, including its cross-sectional nature and reliance on self-report measures, it lays the groundwork for future research in this area. Larger, longitudinal studies are needed to further elucidate the causal relationships between these traits and PLEs, as well as to investigate potential protective factors and intervention strategies.

In conclusion, this research contributes to our understanding of the interplay between neurodevelopmental traits and psychotic experiences in young people. It emphasizes the need for a nuanced, integrated approach to mental health assessment and treatment, particularly during the critical developmental periods of adolescence and young adulthood. Future research and clinical practice should continue to explore these relationships to improve early identification and intervention strategies for at-risk individuals, and future studies may clarify potential mediators of these associations, such as stressful life events.

## Figures and Tables

**Table 1:** Socio-demographic characteristics of the sample (N = 57).

Variable	Mean ± SD (Range), or N (%)
Age at referral	18.26 ± 2.06
Gender	
Male	18 (31.6)
Female	37 (64.9)
Non-binary	2 (3.5)
Educational level	
Middle school	32 (56.1)
High school	25 (43.9)
Employment	
Student	44 (77.2)
Worker	4 (7)
Student/worker	5 (8.8)
Unemployed	4 (7)
Living status	
Alone	6 (10.5)
With family	51 (89.5)
Marital status	
Never married	57 (100)
Offspring	0 (0)

**Table 2:** Clinical characteristics of the sample (N = 57).

Variable	Mean ± SD (Range), or N (%)
Diagnosis	
Anxiety disorders	37 (64.0)
Personality disorders	11 (19.3)
Affective disorders	9 (15.8)
ASRS total	3.19 ± 1.86
AQ-10 total	4.12 ± 2.1
Cape total Frequency	2.16 ± 0.48
Cape total Distress	2.39 ± 0.66
Cape Positive Frequency	1.77 ± 0.46
Cape Positive Distress	2.07 ± 0.7
Cape Depressive Frequency	2.74 ± 0.7
Cape Depressive Distress	2.98 ± 0.75
Cape Negative Frequency	2.39 ± 0.62
Cape Negative Distress	2.56 ± 0.82

**Table 3:** Associations between ASRS and AQ-10 scores and CAPE scores (beta, 95% CI, variance explained). The model included ASRS, AQ-10, age, and gender as independent variables and CAPE total and subscores as dependent variables.

		Cape_Tot_Freq Beta (95% CI)	Cape_Tot_Distress Beta (95% CI)	Cape_Pos_Freq Beta (95% CI)	Cape_Pos_Distress Beta (95% CI)
**ASRS**	β	0.47	0.50	0.36	0.48
	C.I.	(0.06–0.18)	(0.09–0.25)	(0.02–0.16)	(0.09–0.27)
	R^2^	0.49 **	0.40 **	0.26 *	0.38 **
**AQ-10**	β	0.45	0.34	0.31	0.32
	C.I.	(0.05–0.15)	(0.03–0.18)	(0.01–0.13)	(0.03–0.19)
	R^2^	0.49 **	0.40 *	0.26 *	0.38 *
		**Cape_Dep_Freq** **Beta (95% CI)**	**Cape_Dep_Distress** **Beta (95% CI)**	**Cape_Neg_Freq** **Beta (95% CI)**	**Cape_Neg_Distress** **Beta (95% CI)**
**ASRS**	β	0.46	0.55	0.43	0.51
	C.I.	(0.08–0.25)	(0.12–0.32)	(0.06–0.22)	(0.11–0.33)
	R^2^	0.47 **	0.40 **	0.44 **	0.37 **
**AQ-10**	β	0.42	0.26	0.46	0.28
	C.I.	(0.06–0.21)	(0.01–0.18)	(0.07–0.20)	(0.01–0.20)
	R^2^	0.47 **	0.40 *	0.44 **	0.37 *

Community Assessment of Psychic Experiences (CAPE); Adult ADHD Self-Report Scale (ASRS); Autism Spectrum Quotient—short form (AQ-10). * *p* < 0.05; ** *p* < 0.001.

## Data Availability

The original contributions presented in the study are included in the article; further inquiries can be directed to the corresponding authors.
